# Heterozygous Knockout of *ARID4B* Using CRISPR/Cas9 Attenuates Some Aggressive Phenotypes in a Breast Cancer Cell Line

**DOI:** 10.3390/genes14122184

**Published:** 2023-12-06

**Authors:** Fernando Gonzalez-Salinas, Jessica Herrera-Gamboa, Rocio Rojo, Victor Trevino

**Affiliations:** 1 Tecnologico de Monterrey, Escuela de Medicina y Ciencias de la Salud, Ave. Morones Prieto 3000, Monterrey 64710, Nuevo Leon, Mexico; fergzzsal@gmail.com (F.G.-S.); dra.jessica.herrera@gmail.com (J.H.-G.); rocio.rojo@tec.mx (R.R.); 2 Instituto de Biotecnología, Facultad de Ciencias Biológicas, Universidad Autónoma de Nuevo Leon, San Nicolas de los Garza 66455, Nuevo Leon, Mexico; 3 Tecnologico de Monterrey, Escuela de Medicina y Ciencias de la Salud, Mexico City 14380, Mexico; 4 Tecnologico de Monterrey, The Institute for Obesity Research, Eugenio Garza Sada Avenue 2501, Monterrey 64849, Nuevo Leon, Mexico; 5 Tecnologico de Monterrey, oriGen Project, Eugenio Garza Sada Avenue 2501, Monterrey 64849, Nuevo Leon, Mexico

**Keywords:** *ARID4B*, mutations, CRISPR/Cas9, breast cancer, proliferation, RNA-seq

## Abstract

Breast cancer is one of the leading causes of death in women around the world. Over time, many genes and mutations that are associated with the development of this disease have been identified. However, the specific role of many genes has not yet been fully elucidated. Higher *ARID4B* expression has been identified as a risk factor for diverse cancer types. Silencing experiments also showed that *ARID4B* is associated with developing cancer-associated characteristics. However, no transcriptomic studies have shown the overall cellular effect of loss of function in breast cancer in humans. This study addresses the impact of loss-of-function mutations in breast cancer MCF-7 cells. Using the CRISPR/Cas9 system, we generated mutations that caused heterozygous truncated proteins, isolating three monoclonal lines carrying insertions and deletions in *ARID4B*. We observed reduced proliferation and migration in in vitro experiments. In addition, from RNA-seq assays, a differential expression analysis shows known and novel deregulated cancer-associate pathways in mutated cells supporting the impact of *ARID4B.* For example, we found the AKT-PI3K pathway to be altered at the transcript level but through different genes than those reported for *ARID4B*. Our transcriptomic results also suggest new insights into the role of *ARID4B* in aggressiveness by the epithelial-to-mesenchymal transition and TGF-β pathways and in metabolism through cholesterol and mevalonate pathways. We also performed exome sequencing to show that no off-target effects were apparent. In conclusion, the *ARID4B* gene is associated with some aggressive phenotypes in breast cancer cells.

## 1. Introduction

Breast cancer (BC) has the highest global incidence in women [1]. Up to 16% of women worldwide die every year from causes related to this disease [2]. Similarly to other types of cancer, BC is a genetic disease characterized by the presence of genomic alterations [3] that can be either inherited or acquired throughout life [4]. Only a reduced set of genomic alterations identified so far trigger cellular transformations that lead to the onset of cancer and tumor growth [5,6]. However, many genes still require thorough analysis to unravel their potential key role in tumor development and establishing high-risk cancer phenotypes.

*ARID4B*, also known as RBBP1-like protein, is a member of the ARID (AT-rich Interaction Domain) family, which modulates the activity of genes involved in cell proliferation and chromatin remodeling [7]. The Human Protein Atlas and previous studies of *ARID4B* in normal tissues show transcriptional levels in the testes, brain, skin, gallbladder, and, to a minor extent, the thymus, prostate, and ovary [8,9]. In addition, *ARID4B* and *ARID4A* have been found to regulate PWS/AS (Prader–Willi syndrome and Angelman syndrome), indicating that they may play an important role in epigenetic mechanisms [10].

We noted from a previous study that *ARID4B* mutations are related to a reduced survival time [11] and that other members of the ARID family are well-studied in BC. For example, *ARID1A* and *ARID1B* are known to be driver genes in BC [12], while mutations in estrogen receptor-positive BC show a higher risk [13]. Moreover, low expression of *ARID1A* is associated with a more aggressive phenotype [14]. The high expression of *ARID1B* is also associated with a higher histological grade and tumor size [15]. In addition, the silencing of this gene in triple-negative cells produces a delay in the cell cycle [15], indicating that different members of the ARID family could be associated with better or worse prognosis in cancer, suggesting that differences in domains and protein sequences lead to different outcomes. Thus, we focus on *ARID4B,* whose mutations have not been studied deeply.

Transcriptional upregulation of *ARID4B* has been detected in various human carcinomas. In hepatocellular carcinoma, levels of *ARID4B* show a positive correlation with tumor progression and adverse prognosis [16]. In prostate cancer, especially in the PTEN-null phenotype, upregulation of *ARID4B* activates the PTEN-PI3K pathway through specific transactivation of both *PIK3CA* and *PIK3R2*, which favors tumor progression and correlates positively with prostate cancer recurrence [17]. Contrary, in human leukemias, *ARID4B* seems to exert a tumor suppressor activity, and either its downregulation or heterozygous inactivating mutations alongside mutations in *ARID4A* in vivo lead to leukemic transformation through indirect epigenetic mechanisms [18]. In mice, *ARID4B* expression has been found to promote the proliferation and invasiveness of malignant cells; in fact, expression increases tumor growth in vivo and in vitro [19]. In addition, variations in miR-290, which targets *ARID4B*, have been validated in in vitro models, reducing proliferation and metastasis [20]. Using databases of cancer-related hotspots, we have found that the *ARID4B* gene has two hotspots that have never been validated [21].

Overall, the above evidence shows that *ARID4B* has an important role in BC and observed recurrent mutations of *ARID4B* in BC patients suggest a possible impact. However, there are no previous analyses about the effects of *ARID4B* loss-of-function in human cells. In this work, we generated loss-of-function mutations by targeting *ARID4B* in MCF-7 cells using the CRISPR/Cas9 system in one of these critical hotspots previously found. After validation by targeted and exome sequencing, we evaluated how the disruption of *ARID4B* impacts the viability and functionality of BC cells by analyzing cell proliferation, viability, and migration. Furthermore, an expression analysis using RNA-seq in MCF-7 cells with loss-of-function mutations in *ARID4B* compared to the wild-type shows the transcriptional effect of these mutations. Our results shed light on the importance of *ARID4B* mutations in cancer.

## 2. Materials and Methods

### 2.1. Expression and Survival Analysis

Information related to expression levels of *ARID4B* in breast cancer and normal human tissues was obtained from TCGA (The Cancer Genome Atlas) data using the Gene Expression Profiling Interactive Analysis (GEPIA) platform [22], available at http://gepia.cancer-pku.cn/detail.php?gene=ARID4B (accessed on 21 October 2022). TCGA information about survival analyses of breast cancer patients and normal human tissues was obtained using The University of Alabama at Birmingham Cancer Data Analysis Portal (UALCAN) [23], available at http://ualcan.path.uab.edu (accessed on 7 February 2023). Analysis of the effects exerted by mutations in *ARID4B* on the survival of BC patients was carried out on TCGA data using the tool VALORATE (Velocity and Accuracy of the Log-Rank Test) [11]. All figures obtained from these platforms were adapted for this manuscript. Identification of recurrent mutations in the *ARID4B* sequence was carried out using the HotspotAnnotations Database [21].

### 2.2. Cell Culture and Transfection

Protocols for culture and transfection of MCF-7 cells have been described previously [24]. Briefly, MCF-7 cells were cultured at 37 °C, 95% humidity, and 5% CO_2_ in a culture medium containing DMEM-F/12 (Gibco; Thermo Fisher Scientific, Inc., Waltham, MA, USA) supplemented with 10% fetal bovine serum (Invitrogen; Thermo Fisher Scientific, Inc., Waltham, MA, USA) and 1% penicillin/streptomycin (Invitrogen; Thermo Fisher Scientific, Inc.). Cells were cultured at 70% of confluence in 6-well plates and were then transfected with 4 µg of pX459 vector or pX459-*ARID4B* construct using 6 µL of XtremeGene9 Transfection Reagent (Sigma-Aldrich; Merck KGaA, Darmstadt, Germany) and 200 µL of Optimem Medium (Gibco; Thermo Fisher Scientific, Inc.). Plasmid pX459 was a gift from Feng Zhang (Addgene plasmid # 62988; http://n2t.net/addgene:62988; RRID:Addgene_62988) [25]. After 48 h, medium was replaced, and fresh medium with 6 µg/mL of puromycin (Sigma-Aldrich; Merck KGaA) was added. Again, after 48 h, when cells in the controls were all dead, the selection was finished, and fresh medium was added to recover and grow the cells for one week.

### 2.3. sgRNA Design and CRISPR/Cas9 System Construction

sgRNA sequences for CRISPR/Cas9 were designed using the CRISPR design tool (previously available at http://crispr.mit.edu/ (accessed on 23 June 2017) provided by the Feng Zhang Lab. These sgRNA targets near the amino acid 939 in the protein Arid4b, an important hotspot found in HotspotAnnotations Data Base. The sequences of this sgRNA are 5′-CACCGCCAGACATCTTTTCGATCTT-3′ (top) and 5′-AAACAAGAT CGAAAAGATGTCTGGC-3′ (bottom). Both oligos were aligned and cloned in Bbs1 digested pX459 (62988, Addgene, Cambridge, MA, USA). Then, we expanded the pX459-ARID4B vector using calcium-competent *Escherichia coli* DH5α. Correct insertion of sgRNA into the pX459 vector was confirmed using Sanger sequencing.

### 2.4. Isolation of Transfected Clones

Single-cell derived colonies were obtained through limiting dilution of cell pools that had been previously transfected with the pX459-ARID4B vector and enriched upon exposure to puromycin. For this, confluent cell pools were trypsinized and counted using Trypan blue (Gibco; Thermo Fisher Scientific, Inc.). Cell suspensions were serially diluted using DMEM/F12, and cell density was adjusted to 5 cells/mL. A total of 100 µL of this solution was transferred to each well of a 96-well plate, and single cells were incubated under standard conditions. Then, 3 days after cell seeding, 96-well plates were inspected through the microscope, and wells containing single cells were selected for further experimentation. Cells were genotyped by Surveyor assay and Sanger sequencing. One of the clones that resulted negative for genetic editions in ARID4B was used as negative control (NC).

### 2.5. Validation of Loss-of-Function Mutations

Isolation of DNA from transfected monoclonal cells was carried out using the GenElute^TM^ Mammalian Genomic DNA Miniprep Kit (Sigma-Aldrich; Merck KGaA). Amplification of an 838 bp region of *ARID4B* was performed by PCR, using the GoTaq^®^ DNA Polymerase (Promega Corporation, Madison, WI, USA) with the following primers: 5′- GCTGAAGACAGTGAGCAGGA-3′and 5′-CGACATTGACTGGAGGTGGT-3′. The presence of mutations in the amplified genomic region was confirmed through the Surveyor assay. For this, PCR amplicons were denatured and reannealed for heteroduplex formation. DNA heteroduplexes were processed using the Surveyor^®^ Mutation Detection Kit (IDT, Coralville, IA, USA). DNA fragments obtained upon enzyme-mediated detection of mismatching nucleotides and cleavage of DNA sequences were separated through agarose gel electrophoresis. Clones carrying the correct genomic modification at *ARID4B* were detected through Sanger sequencing using the BigDye Terminator v1.1 Cycle Sequencing Kit (Invitrogen, Thermo Fisher Scientific, Inc.) as per manufacturer’s instructions. Briefly, a master mix containing 1 µL of Big Dye Terminator and 2 µL of Buffer 5X was mixed with 3.2 pmol of primer 5′-GCTGAAGAGAGTGAGCAGGA-3′ and 20 ng of purified PCR product. Then, the reaction volume was adjusted to 10 µL using nuclease-free water. Amplified products were purified using the BigDye X-Terminator Purification Kit (Invitrogen, Thermo Fisher Scientific, Inc.) and analyzed using the ABI PRISM 3100 Genetic Analyzer (Applied Biosystems; Thermo Fisher Scientific, Waltham, MA, USA). Sequences were analyzed using the INDIGO platform [26] at https://gear.embl.de/indigo/ (accessed on 28 October 2018) to detect mutations. 

### 2.6. Whole Exome Sequencing and Analysis of Off-Target Effects

Whole-Exome Sequencing (WES) was carried out as follows. First, DNA was isolated from cells as described previously. Isolated DNA was quantified using the Qubit™ dsDNA H.S. Assay Kit (Invitrogen, Thermo Fisher Scientific, Inc.) and the Qubit 4 fluorometer. Exome sequencing was performed using the Illumina HiSeq 2000 sequencer (Illumina, San Diego, CA, USA) via a paired-end for 100-bp protocol following the manufacturer’s instructions. Reads were mapped to the reference human genome (hg38) using the BWA-MEM alignment algorithm [27] and visualized using the Integrative Genomics Viewer (IGV) program [28]. Post-processing of data was carried out using the Galaxy web platform [29]. Potential off-target effects of the selected sgRNAs were analyzed using the CRISPR-Cas9 guide RNA design checker developed by IDT, available at https://www.idtdna.com/site/order/designtool/index/CRISPR_SEQUENCE (accessed on 23 January 2023); and genes with a higher score for potential off-target effects (*ZNF615*, *TOP2B*, *LACTB*, and *LDLRAP1*) were analyzed through WES. BioProject accesion number in NCBI of WES data is PRJNA930489 https://www.ncbi.nlm.nih.gov/sra/PRJNA930489. 

### 2.7. Expression Analysis

Total RNA was isolated from clones using GenElute™ Mammalian Genomic DNA Miniprep Kit (Sigma-Aldrich; Merck KGaA). The integrity of RNA was analyzed through gel electrophoresis. cDNA was synthesized from 1 µg of purified RNA using the High-Capacity cDNA Reverse Transcription Kit (Invitrogen; Thermo Fisher Scientific, Inc.). qPCR reactions were prepared using the PowerUp™ SYBR^®^ Green Master Mix (Invitrogen; Thermo Fisher Scientific, Inc.) and primers: 5′-GGCCCACTAAAGGTAGGAGC-3′ and 5′-AGTGTACCAACTCGCATCTGT-3′). *GAPDH* was used as a housekeeping gene to normalize Ct values, using primers: 5′-GAGTCAACGGATTTGGTCGT-3′ and 5′-TTGATTTTGGAGGGATCTCG-3′). The relative expression of each amplicon was analyzed by the 2^−ΔΔCt^ method [30].

### 2.8. Cell Growth Analysis

A total of 2 × 10^3^ cells were seeded in each well of 96-well plates containing 90 µL of supplemented DMEM/F12. Following 24 h of incubation, 10 µL of Alamar Blue reagent (Invitrogen; Thermo Fisher Scientific, Inc.) was added to each well, and cells were incubated for 4 h. Then, the fluorescence signal was acquired at 530 nm and 590 nm for excitation and emission wavelengths, respectively. Cell proliferation was measured at 24, 48, and 72 h, using CyQUANT^®^ Direct Cell Proliferation Assay (Invitrogen; Thermo Fisher Scientific, Inc.), following the manufacturer’s instructions. In both cases, samples were read using a Bio-Tek Synergy HT Multi-Detection Microplate Reader (Bio Tek Instruments, Inc., Winooski, VT, USA).

### 2.9. Cell Migration Assay

The migration of cells was tested using the in vitro scratch assay [31]. Briefly, 24-well plates were seeded with 2.4 × 10^5^ cells per well in triplicates. After overnight incubation under standard conditions, the attachment of cells was confirmed through observation under the microscope. Then, a region of attached cells was cleared in each well through manual scratch, using a 200 µL pipet tip, washing with PBS, and adding fresh culture medium. The area corresponding to the induced wound was identified through the microscope and marked as a reference for future measurements. Plates were incubated for 24 h. Then, the area corresponding to the induced scratch (i.e., the wound) was photographed under a light microscope and analyzed using Image J. Cell migration was determined as the percentage of the area corresponding to the induced wound that had been covered by migrating cells after 24 h of incubation.

### 2.10. RNA-seq and Enrichment Analysis

RNA was isolated from the clones carrying genomic modifications at *ARID4B* and negative control cells (NC). RNA integrity was verified using agarose gel and a Nanodrop instrument. RNA libraries were prepared using 300 ng of total RNA and the TruSeq Stranded mRNA Sample Preparation Kit (Illumina, San Diego, CA, USA). The quality of the libraries was verified by the Qubit fluorescence instrument and protocols. Sequencing was carried out using a MiSeq Reagent Kit v3 in a MiSeq Sequencer (Illumina, San Diego, CA, USA), as instructed by manufacturer. A total of 14 million reads passing filter, 76 × 2 cycles, pair-end, 91% Q30, and 50–52% GC content were obtained for the 4 cell types (3 edited clones and negative control), averaging 3.6 M reads per cell type and a minimum of 2.9 M reads. Processing of RNA-seq data was carried out using the Galaxy web platform [32]. Quality control was performed using FastQC (https://www.bioinformatics.babraham.ac.uk/projects/fastqc/). Reads were trimmed using TRIMMOMATIC [33] and mapped using HiSAT2 [34] using hg38 as the human reference genome and default parameters. Read counts were then obtained with FeatureCounts [35]. Because genetic editions are minor, differences in gene expression were expected to be subtle; for that reason, differential gene expression was defined as a 1-fold difference (fold-change defined as log_2_(A/B)) in samples that had more than 20 reads, following library size (read count) normalization and logarithm transformation processed in the R language (https://cran.r-project.org/). Transcriptomic data are available in GEO at NCBI with the accession number GSE224514 https://www.ncbi.nlm.nih.gov/geo/query/acc.cgi?acc=GSE224514. Functional enrichment analysis was carried out using ENRICH R [36], considering only functional terms with a Benjamini–Hochberg FDR-adjusted-*p*-value (*q*-value) < 0.1 (10% FDR) and overlapping with more than three genes.

### 2.11. Statistical Analysis

Data were analyzed using GraphPad and Prism 5. Student’s *t*-test was used to assess differences. A value of *p* < 0.05 was considered statistically significant in all the experiments. When apply * indicates *p* < 0.05, ** indicates *p* < 0.005, *** indicates *p* < 0.0005. Principal Components Analysis (PCA) was performed in the R language using the prcomp() function.

## 3. Results

### 3.1. ARID4B Expression and Mutations Are Associated with Survival in BC Patients

**Figure 1:**
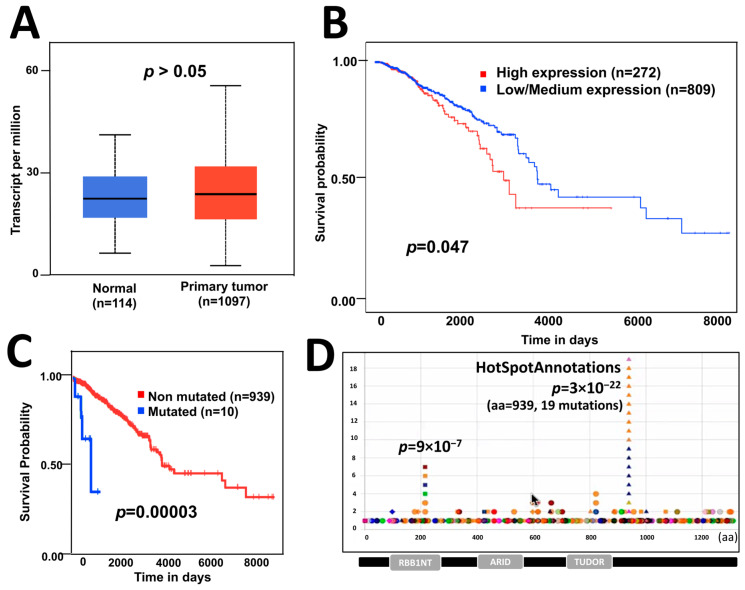
**The effect of *ARID4B* expression and mutations on patient survival.** (**A**) Expression of *ARID4B* is similar in patients with breast cancer compared with normal tissue, from GEPIA data. (**B**) High expression of *ARID4B* is associated with poor survival in patients with breast cancer from TCGA data. (**C**) Point mutations in *ARID4B* are associated with low survival in breast cancer patients using statistical analysis for unbalanced groups (VALORATE). (**D**) Hotspot found in the HotspotAnnotations database to be analyzed in further experiments.

To know the effect of *ARID4B* expression in BC patients, we analyzed data from TCGA and the GEPIA database [22] and compared the expression of *ARID4B* between BC patients and normal individuals. The mean *ARID4B* expression level was not statistically different across groups (Figure 1A). However, BC patients who had higher levels of *ARID4B* expression showed lower survival times compared to those BC patients who had low/mid expression levels of *ARID4B* (Figure 1B). Additionally, we identified the different mutations across the sequence of *ARID4B* to know the survival probabilities of BC patients. Although the group of mutated patients is tiny (*n* = 10), our analysis revealed that patients carrying mutations in the *ARID4B* sequence had significantly lower survival rates compared to patients bearing no mutations in that locus (Figure 1C). To better understand the effect of loss-of-function mutations in the ARID4B gene, we selected the most significant hotspot at the amino acidic position 939 for validation assays according to HotSpotAnnotations (Figure 1D). Although we chose this specific mutation, the effects of frameshifts generated by CRISPR/Cas9 would be similar to those losses of function mutations in DNA positions of T939 and further.

### 3.2. Generation of ARID4B Knock-Out in the MCF-7 Cell Line

**Figure 2:**
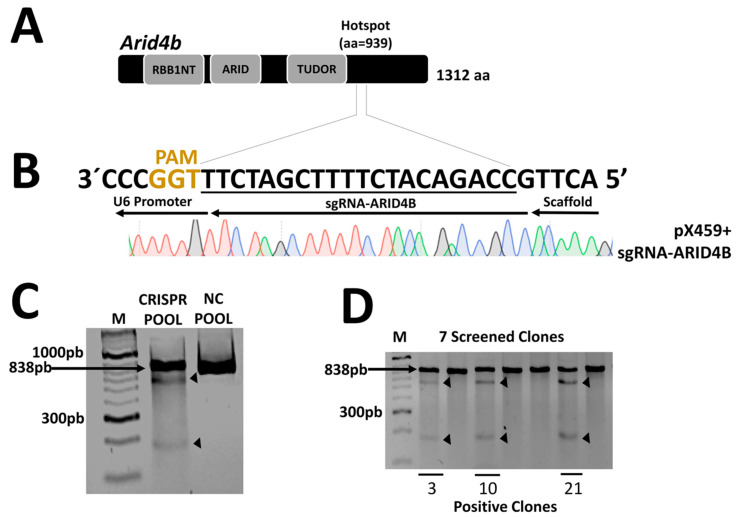
**Construction and validation of the CRISPR/Cas9 system.** (**A**) The structure of the *ARID4B* protein with a total of 1312 amino acids and the site where the mutations were generated in position 939, indicated as a hotspot. (**B**) Design of the sgRNA and validation of the ligation in the pX459 vector by Sanger sequencing. Only underlined region is shown. Red for T, Black for G, Blue for C, and Green for A. (**C**) Confirmation of the presence of genetic editions in the pool of MCF-7 cells after transfection. The surveyor assay produces a cut in the PCR product, generating two fragments indicated by arrows. (**D**) Validation of the presence of genetic editions in isolated clones. Here, 3, 10, and 21 represent the clones positive for mutations in *ARID4B*.

We focus on editing the region of the *ARID4B* hotspot at position 939 (Figure 2A) by using a region-specific sgRNA and the CRISPR/Cas9 system cloned into the pX459 vector. The sequence of the ligated vector was verified by Sanger sequencing (Figure 2B) and transfected into MCF-7 cells. The cell pool was verified using the Surveyor assay by digesting the 838 bp PCR product from the *ARID4B* region. After digestion, the two expected fragments of approximately 628 bp and 210 bp were clearly observed, indicating the edition of the target region (Figure 2C). After the selection of seven monoclonal populations by limiting dilution, three clones carrying the genetic edition of the *ARID4B* region gene were obtained (Figure 2D). From here, these three clones will be named *ARID4B*-3, *ARID4B*-10, and *ARID4B*-21.

### 3.3. Characterization of Genetic Editions in ARID4B-Edited MCF-7 Clones

**Figure 3:**
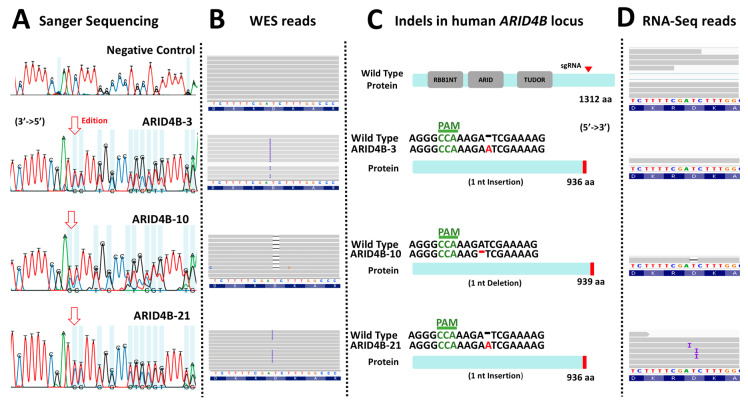
**Characterization of loss-of-function mutations in *ARID4B*.** (**A**) The result of the Sanger sequencing showing altered electropherograms in the three clones previously validated by Surveyor assay. (**B**) Result of the WES analysis of the three clones. Clones *ARID4B*-3 and *ARID4B*-21 have an adenine insertion. On the other hand, clone *ARID4B*-10 has an adenine deletion. (**C**) Characterization of the effect of the mutations found in the three clones. Two of the clones (*ARID4B*-3 and *ARID4B*-21) alter the reading frame, producing a hypothetical truncated protein of 936 amino acids. Clone *ARID4B*-10 would produce a 939 amino acid truncated protein. (**D**) RNA-Seq reads showing that edited DNA have been transcribed in at least two clones (*ARID4B*-10 and *ARID4B*-21).

**Figure 4:**
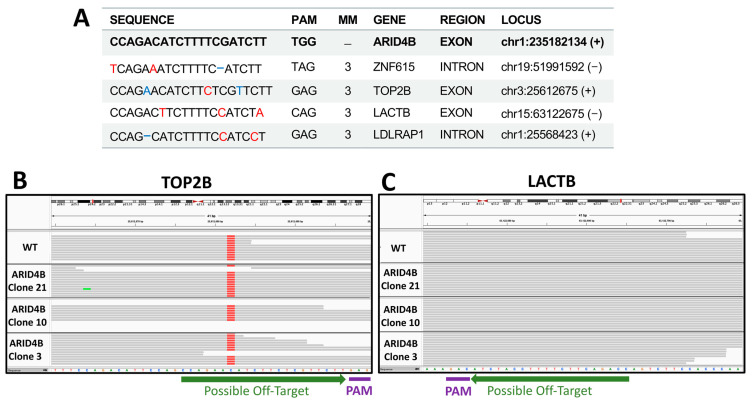
**Off-targets validation.** (**A**) Top possible off-target sequences. Red nucleotides mark mismatches with respect to the ARID4B sequence. Blue positions highlight insertions or deletions. Possible off-targets in exons are shown in B and C. (**B**) Exome sequencing of TOP2A possible off-target region in clones and wild-type cell lines. (**C**) Exome sequencing of LACTB possible off-target region in clones and wild-type cell line. No differences are observed relative to wild type (WT).

To identify the precise genetic edition obtained in the three clones, the region flanking the site of interest in this gene was sequenced using Sanger sequencing (Figure 3A). Electropherograms were characterized using the INDIGO platform to determine the specific mutations. Then, using the reads from the whole exome sequencing assay, we noted that the site of interest was indeed correctly edited as described, as shown in the selected examples (Figure 3B). As a result, it was found that clones *ARID4B*-3 and *ARID4B*-21 had the same heterozygous insertion of adenine that may produce a truncated protein of 936 amino acids compared to the wild-type protein, which has 1312 amino acids. On the other hand, the clone *ARID4B*-10 carries a deletion that also affects the reading frame of this gene, which would produce a truncated protein of 939 amino acids (Figure 3C). This confirms the correct introduction of heterozygous loss-of-function mutations in the three clones producing altered versions of the original protein. We performed WES experiments to evaluate the target and off-target regions further. We prioritized regions listed by the software used (see Section 2), which revealed four genes (ZNF615, TOP2B, LACTB, LDLRAP1, see Figure 4). The off-target region for two of these genes are exons (TOP2B and LACTB) that can be analyzed by our exome sequencing experiment. We did not find any recognized edition around the expected off-target site for these two genes relative to wild-type in the cell line, confirming that the edition was specific for the target site in the *ARID4B* gene (Figure 4). By RNA-Seq, we also confirmed that the alleles carrying genetic editions were indeed transcribed in *ARID4B*-10 and *ARID4B*-21 clones (Figure 3D). In the *ARID4B*-3 clone, we only observed one read of the unedited allele (due to the low number of reads sequenced in that clone). Therefore, the possible functional effects should be attributed to *ARID4B* editions.

### 3.4. MCF-7 Clones Carrying Induced Mutations in ARID4B Show Reduced Cell Proliferation and Viability

**Figure 5:**
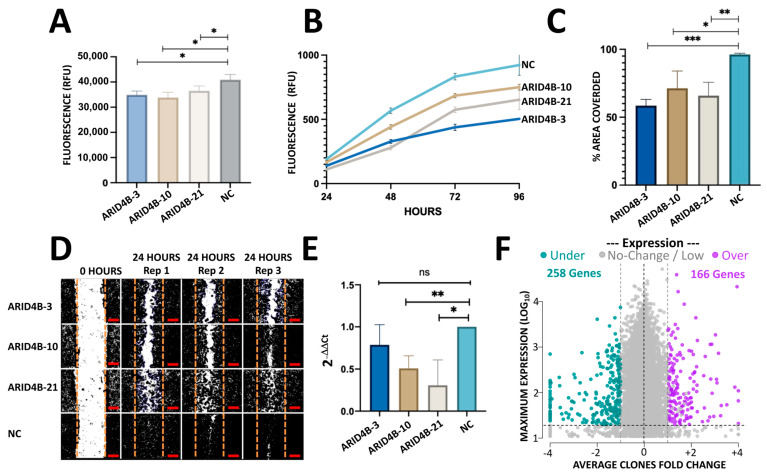
In vitro characterization of MCF-7 cells with mutations in *ARID4B*. (**A**) Viability assay with Alamar Blue shows a reduction in the three clones of *ARID4B* compared with NC. Results are shown as mean ± SEM (n = 3). (**B**) Mutations in *ARID4B* reduce proliferation using CyQuant Proliferation Kit. Results are shown as mean ± SEM (n = 3). (**C**,**D**) Reduction in cell migration in the three clones with mutations in ARID4B in a wound healing assay. Scale bars represent 100 µm. (**E**) qPCR analysis shows a reduction in expression in MCF-7 cells with mutations in *ARID4B*. (**F**) RNA-seq results gave a total of 424 genes up and down-regulated in cells with mutations compared with NC. The horizontal axis shows the average fold change from the 3 clones relative to NC. The vertical axis shows the maximum expression observed from the 3 clones and NC. In all cases * indicates *p* < 0.05, ** indicates *p* < 0.005 and *** indicates *p* < 0.0005.

To determine whether the induced mutation editions in *ARID4B* alter the proliferation and viability of BC cells, we measured the cell growth of clones *ARID4B*-3, *ARID4B*-10, and *ARID4B*-21 at different time points through the two following experiments. First, we used the Alamar Blue colorimetric test. Results show that our *ARID4B* clones are associated with reduced viability in MCF-7 cells at 48 h (*p* < 0.05, Figure 5A). Second, using the Cyquant Proliferation Kit, in which a reduction in cell proliferation was found from 48 to 96 h (*p* < 0.05) in the 3 clones compared with the NC (Figure 5B). Taking the results of both experiments, it was found that the *ARID4B* gene affects cell growth in MCF-7 cells. 

### 3.5. Knockout of ARID4B Affects Migration Capacity

To evaluate whether *ARID4B* plays an essential role in cell migration, we assessed the wound-healing capacity of clones *ARID4B*-3, *ARID4B*-10, and *ARID4B*-21. Specifically, we imaged the area of the culture well that corresponded to the induced wound (which had been scratched with a pipet tip 24 h before imaging); then, we analyzed images using Image J and measured the percentage of area covered by each clone, relative to NC after 1 day of incubation. All three clones carrying mutations in *ARID4B* showed a decreased ability to migrate and cause “wound” closure, compared to NC (*p* < 0.05, Figure 5C,D). These results suggest that the presence of loss-of-function mutations in *ARID4B* is associated with lower migration rates in MCF-7 BC cells, potentially attenuating the metastatic capacity of BC cells.

### 3.6. Induction of Loss-of-Function Mutations Leads to Transcriptional Alterations of ARID4B

Using qPCR, a reduction in *ARID4B* expression levels was found in cells with loss-of-function mutations compared to wild-type cells. Specifically, clone *ARID4B*-21 was the clone that had a lower expression compared to NC, followed by *ARID4B*-3 and *ARID4B*-10 (Figure 5E). The order of expression level is very similar to that observed in RNA-seq data (31.7, 80.5, 121.4 transcripts per million in *ARID4B*-21, -10, and -3 clones, respectively).

### 3.7. Heterozygous Loss-of-Function Mutations in ARID4B Alter the Expression of Several Genes

We analyzed the transcriptome effect of the three clones by RNA-seq experiments. After read-count normalization, averaging across clones, and filtering by minimal counts and +/−1 fold-change in all clones relative to NC, a total of 166 genes were found to be overexpressed and 258 downregulated (Figure 5F). The top 20 genes are shown in Table 1 while the whole results are available in Supplementary Material (raw and processed data, PCA, and Heatmap in Figures S1 and S2). To further investigate the biological functions of these 424 deregulated genes, we performed 2 gene enrichment analyses using EnrichR and configuring different databases such as KEGG, Reactome, Bioplanet, and WikiPathways. The lists of up- and down-regulated genes were analyzed separately. To simplify the associations from different databases, we merged similar concepts and gene content in a “simplified concept”, as shown in Table 2 (see Supplementary Material for raw results). Some of the pathways that are enriched with the up-regulated genes are associated with cancer, such as epithelial to mesenchymal transition in colorectal cancer (FDR = 6%), PI3K-AKT signaling pathway (FDR = 2%) and focal adhesion-PI3K-AKT-mTOR (FDR = 8%). Other general significant pathways are amino acid biosynthesis (FDR < 1%), ECM receptor interaction (FDR = 0.9%), collagen metabolism, cytochrome P450, NCAM, and Platelet Signaling, among others (Table 2).

On the other hand, down-regulated genes are associated with cholesterol biosynthesis (FDR ≤ 1%), metabolism of steroids (FDR ≤ 1%), TGF-β regulation of extracellular matrix (FDR ≥ 0.01%), and target of rapamycin (TOR) (FDR = 3%) (Table 2). These results suggest that *ARID4B* could exert an important role in regulating genes associated with cancer progression.

## 4. Discussion

The ARID gene family is involved in regulating gene transcription and modifying chromatin structure [37]. Some members have been associated with cancer since these genes are frequently mutated in tumors and are related to cancer pathways [38]. In this work, we analyzed *ARID4B* specifically, which is involved in different functions, such as regulating male fertility [39] and spermatogenesis [40]. However, there are several studies related to the role of *ARID4B* and its participation in the development of different types of cancer. For example, in human glioma, it is highly expressed in cancer cell lines compared to normal brain tissue, and the higher expression correlates with WHO grade in patients [7]. On the other hand, *ARID4B* has been associated with tumor promoter functions regulated by microRNAs in prostate cancer [41], and others associate it as a tumor suppressor in leukemias [18]. In breast cancer, studies have been performed on mouse cells in which an ectopic expression of the *ARID4B* gene has been introduced, and an increase in proliferative and migratory capacities has been found. [19]. Furthermore, with the help of knockdown methodologies, a reduction in metastasis has also been demonstrated, which indicates that *ARID4B* would also have an important role in this type of cancer [19]. We did not find differences in the expression of *ARID4B* in breast cancer samples compared to normal tissues (Figure 1A), in contrast to what has already been observed in other types of cancer, such as hepatocellular [16] and human gliomas [42]. However, as previously documented in databases, worse survival was found in patients with a high expression (Figure 1B) [43]. This could indicate that the expression of *ARID4B,* specifically in BC, is associated with a more aggressive phenotype.

On the other hand, we also found that point mutations in *ARID4B* are associated with reduced survival in BC patients (Figure 1C). This evidence suggests that these mutations could be generating an over-activation. We recently found an important hotspot in *ARID4B* position 939 involving four cancer types (uterine, stomach, colorectal, and thymus). The hotspot is characterized by frame shifts that could be related to cancer progression also in BC. Thus, we decided to edit this region to find the effect of loss-of-function mutations generating heterozygous knockouts in MCF-7 cells using the CRISPR/Cas9 system. Our results clearly show that *ARID4B* frame-shift mutations around amino acid 939 reduce aggressiveness. Thus, the mutations observed decreasing survival in BC, which should correspond to gain of functions or loss-of-function before hotspot 939. 

Since the function of *ARID4B* seems important in several cancer types, this study aimed to analyze the effect of loss-of-function in human breast cancer cells and to evaluate the pathways deregulated by the impact of the loss-of-function of the *ARID4B* gene. Our in vitro experiments revealed decreased viability, proliferation, and migration in clones with loss-of-function mutations compared to the negative control. Similar results have been found in other types of cancer, such as prostate and glioma mentioned above. For this reason, this study supports the hypothesis that the *ARID4B* gene is associated with promoting cancer-associated characteristics. Therefore, it would be interesting to observe other mutations that could explain the clinical observation.

Of the three clones obtained, *ARID4B*-3 and *ARID4B*-21 have a deletion, which would produce a truncated 939 amino acidic protein, while clone *ARID4B*-10, on the other hand, has an insertion that could produce a 936 amino acidic protein. Even though there is only a putative difference of three amino acids, the phenotypic assays revealed some differences between both types of mutations. The *ARID4B*-10 clone revealed a more significant negative effect on proliferation and migration. In contrast, the other two clones had similar results in both experiments. We noted an agreement in migration and proliferation across clones, suggesting that migration could be influenced by proliferation. In all cases, the mutations produced were heterozygous (partial knockout), which seems more similar to the genomic context in breast cancer patients in comparison to previous experiments performed in *ARID4B* that used RNA silencing or knockouts deleting both alleles and cell lines other than breast-derived [17,19,42]. In this way, the results of the in vitro characterization could explain the behavior of cells in BC development in a more specific manner.

In a systematic review, we have reported that the MCF-7 cell line is the most widely used model in breast cancer, studying mutations by CRISPR/Cas9 [44]. MCF-7 represents Luminal A type of breast cancer [45]. However, there are other cell lines that could display different behavior in breast or other cancer types. Thus, our analysis shows a fraction of the effects that *ARID4B* mutations could develop.

Employing RNA-Seq, the genes deregulated on edited cells were analyzed. We found the AKT-PI3K signaling through diverse collagens, RELN, LPAR5, GNG7, and ITGA5. AKT-PI3K is a critical pathway related to EGFR that regulates proliferation and cell survival [46]. The AKT-PI3K pathway has been previously linked with *ARID4B* in prostate cancer [17] and glioma [42] including downregulation of PI3KCA and PI3KR2. In contrast, we observed downregulation in PI3KC2A, PI3KC2B, PI3KC2G, and PI3KR3 (fold changes −1.6, −1.6, −5.4, and −1.2, respectively). Thus, our lesser cell proliferation agrees with other models but through different genes of the pathway, confirming and expanding our knowledge of the functional effects of *ARID4B* loss of function. 

We also found pathways associated with the epithelial-to-mesenchymal transition (EMT), an important program that promotes the invasiveness of cells and leads to metastasis [47], and the EMT receptor interaction pathway, which has a critical role in cancer cells movement, adhesion, and hyperplasia [48]. Because other genes in the ARID family have been related to EMT pathways, for example, *ARID1A* [49] and *JARID1B* [50], our results suggest that *ARID4B* also has a role in modulating EMT. Its involvement could explain the differences in the migratory capacity found in the wound healing assay in this study and the metastasis observed in in vivo experiments published before [19]. 

Among the down-regulated pathways, two of the most enriched were cholesterol metabolism and the mevalonate pathway. Although no previous studies link *ARID4B* and these pathways, another ARID family member, *ARID1A,* showed an association by knockout experiments [51]. Comparatively, we noted similar genes downregulated such as *IDI1, SQLE, HMGCS1, CYP51A1, DHCR24, MVD, HMGCR,* and *LSS* (fold changes −1.7, −1.9, −2.3, −1.3, −1.2, −2.1, −1.4 and −2.0, respectively). This result suggests that both or more members of the ARID family are involved in regulating these pathways.

Another important altered pathway was the TGF-β regulation, which has been previously implicated with aggressive characteristics of breast cancer modulated by long non-coding RNAs [52,53,54]. In fact, blocking TGF-β can be very useful in developing therapies to inhibit tumor growth and metastasis [55]. *ARID1A* knockdown in MMNK-1 cells results in deregulated TGF-β members and increased proliferation and colony formation [56]. Thus, the expression or function of *ARID1A* and *ARID4B* genes seem to have opposite associations in cancer development. However, it is striking that the deregulation of the TGF-β signaling pathway also produces opposite results. This is consistent with other studies where TGF-β signaling in early cancer stages has a tumor suppressor effect, while in late stages, it has a promoter effect in proliferation and metastasis [55]. Here, using MCF-7 cells, the loss of function of *ARID4B* seems to deregulate genes of the TGF-β signaling pathway in both up- and down-regulated genes, which, by our functional assays, seems consistent with a reduction of aggressiveness. A study to evaluate the loss of function of *ARID1A* and *ARID4B* in both normal and cancer cells is necessary to verify specific effects on the TGF-β pathway, such as adding exogenous TGF-β in a rescue study.

A potential limitation of this study was the lack of funding to perform experiments analyzing Arid4b peptide expression. Although we showed enough evidence of mutated alleles being transcribed and that there is a reduction of gene expression, protein experiments could provide information on the relative amounts of allele-specific peptides and perhaps support the observed differences in clones 3 and 21. In addition, since results show an intriguing agreement in proliferation and migration, with the migration assay used, we can not rule out the influence of proliferation in the migration assays. Thus, more specific assays need to be performed to bring light to the specific role of ARID4B in these two processes.

## 5. Conclusions

The present study demonstrated that loss-of-function mutations close to a frequently mutated position of the *ARID4B* gene diminish its expression and alter the transcriptome program, reducing some aggressive characteristics of breast cancer, such as proliferation, viability, and migration. Therefore, it is likely that somatic mutations observed in patients showing worse prognosis and different frameshifts around amino acid 939 may confer more aggressive features, which should be demonstrated in further experiments. In addition, the RNA-Seq analysis performed in *ARID4B* clones revealed 410 dysregulated genes affecting different pathways, some associated with cancer and metastasis.

## Figures and Tables

**Table 1:** List of top 20 up- and down-regulated genes from RNA-seq analysis.

Gene	Name/Function	Folds
RASAL1	Ras Protein Activator Like 1	6.5
SPINK5	Serine Peptidase Inhibitor Kazal Type 5	5.1
DNAJA4	Dnaj Heat Shock Protein Family Member A4	4.8
CASR	Calcium Sensing Receptor	4.6
RCN3	Reticulocalbin 3	4.3
CYP3A7-CYP3A51P	Cyp3a7-Cyp3a51p Readthrough	4.2
PENK	Proenkephalin	4.2
INHBE	Inhibin Subunit β E	4.0
	LOC101929415	3.9
ZNF532	Zinc Finger Protein 532	3.7
GSTM5	Glutathione S-Transferase Mu 5	3.7
ARMCX5	Armadillo Repeat Containing X-Linked 5	3.6
EPHA7	Eph Receptor A7	3.6
PSG5	Pregnancy Specific β-1-Glycoprotein 5	3.5
ARL11	Adp Ribosylation Factor Like Gtpase 11	3.5
PLXNA2	Plexin A2	3.4
LRRC17	Leucine Rich Repeat Containing 17	3.2
PDE1B	Phosphodiesterase 1b	3.2
HES7	Hes Family Bhlh Transcription Factor 7	3.1
WNT7A	Wnt Family Member 7a	3.1
SNX31	Sorting Nexin 31	−10.6
MEDAG	Mesenteric Estrogen Dependent Adipogenesis	−10.1
CXCL12	C-X-C Motif Chemokine Ligand 12	−8.9
FUCA2	α-L-Fucosidase 2	−8.8
HTATIP2	Hiv-1 Tat Interactive Protein 2	−8.5
TWIST1	Twist Family Bhlh Transcription Factor 1	−8.3
NRCAM	Neuronal Cell Adhesion Molecule	−8.3
TAL1	Tal Bhlh Transcription Factor 1, Erythroid Differentiation Factor	−8.2
TSPYL5	Tspy Like 5	−8.2
ADORA2B	Adenosine A2b Receptor	−8.1
ITIH5	Inter-α-Trypsin Inhibitor Heavy Chain 5	−8.1
AMPH	Amphiphysin	−8.1
TINAGL1	Tubulointerstitial Nephritis Antigen Like 1	−8.1
GSPT2	G1 To S Phase Transition 2	−8.0
CYYR1	Cysteine And Tyrosine Rich 1	−8.0
F2R	Coagulation Factor Ii Thrombin Receptor	−7.9
SLC40A1	Solute Carrier Family 40 Member 1	−7.8
P3H3	Prolyl 3-Hydroxylase 3	−7.7
UAP1L1	Udp-N-Acetylglucosamine Pyrophosphorylase 1 Like 1	−7.6
ADGRG2	Adhesion G Protein-Coupled Receptor G2	−7.4

**Table 2:** List of up-regulated and down-regulated pathways obtained from RNA-seq analysis.

Simplified Concept *	Genes	Lowest q-Value	Highest Odds Ratio	B	R	W	K
↑ AGE-RAGE signaling pathway	6	0.0103	7.9				1
↑ Amino acid metabolism	6	0.0005	40.8	2			
↑ Axon guidance	10	0.0118	4.0	1			
↑ β-1/3 Integrin interactions	6	0.0009	12.4	2			
↑ Collagen metabolism	7	0.0002	69.9	7	4		
↑ Cytochrome P450	5	0.0093	24.5	2	3		
↑ Developmental biology	13	0.0018	4.1	1			
↑ ECM-receptor interaction	6	0.0087	9.0	1			1
↑ Epithelial To Mesenchymal Transition In Colorectal Cancer	7	0.0456	5.7			1	
↑ Extracellular matrix organization	5	0.0234	7.0	1			
↑ Human papillomavirus infection	11	0.0087	4.3				1
↑ NCAM Signaling	5	0.0012	15.8	2	2		
↑ NGF-stimulated Transcription	4	0.0217	14.0		2		
↑ PI3K-Akt signaling pathway	10	0.0320	3.6			1	1
↑ Platelet Signaling and Homeostasis	6	<0.0001	68.4	3			
↑ Prolactin regulation of apoptosis	5	0.0528	5.4	1			
↑ TGF-β regulation of extracellular matrix	19	0.0001	4.6	1			
↓ BDNF signaling pathway	13	0.0031	4.2	1			
↓ Cholesterol Metabolism	11	<0.0001	63.1	1	3	7	
↓ Metabolism Of Steroids	4	0.0012	6.1		1		
↓ Oncostatin M	16	0.0005	4.4	1			
↓ Mevalonate/Terpenoid metabolism	4	0.0023	18.6	1		1	1
↓ Target Of Rapamycin Signaling	4	0.0332	9.7			1	
↓ TGF-β regulation of extracellular matrix	27	<0.0001	4.2	1			
↓ Steroid biosynthesis	5	0.0023	18.6	1			1

Columns *B*, *R*, *W*, and *K* refer to databases Bioplanet, Reactome, Wikipathways, and KEGG, respectively, from the EnrichR database collection. Lowest q-Value and Highest Odds Ratio columns refer to the lowest adjusted *p*-value and highest odds ratio from the set of B + R + W + K gene sets or concepts. ↑ means up-regulation and ↓ means down-regulation. * Simplified concept is a summarized name given by similar concepts from different databases. For example, the concept “Collagen metabolism” in row 5 is a consensus from 7 and 4 concepts from Bioplanet and Reactome, respectively. The full results from EnrichR are provided as supplementary material.

## Data Availability

Whole exome sequencing data is available in BioProject with the accession number PRJNA930489 https://www.ncbi.nlm.nih.gov/sra/PRJNA930489. Transcriptomic data is available in GEO at NCBI with the accession number GSE224514 https://www.ncbi.nlm.nih.gov/geo/query/acc.cgi?acc=GSE224514.

## Supplementary Materials

The following supporting information can be downloaded at: https://www.mdpi.com/article/10.3390/genes14122184/s1, Figure S1: PCA analysis of RNA-seq results; Figure S2. Heatmap of down-regulated and up-regulated genes from RNA-seq analysis; Table S1: Raw data of RNA-seq results; Table S2: Full enrichment analysis results using EnrichR.
