# Supplementary material for: Heterozygous Knockout of ARID4B Using CRISPR/Cas9 Attenuates Some Aggressive Phenotypes in a Breast Cancer Cell Line
Source: Genes (Basel). 2023 Dec 6;14(12):2184. doi: 10.3390/genes14122184 (PMC10743217; doi:10.3390/genes14122184)
Supplement: Supplementary file 1 [file genes-14-02184-s001.zip › Supplementary Figures ARID4B.pdf]

Supplementary Figures for the manuscript

Heterozygous knockout of *ARID4B* using CRISPR/Cas9 attenuates some aggressive phenotypes in a breast cancer cell line.

Fernando Gonzalez-Salinas , Jessica Herrera-Gamboa, Rocio Rojo, and Victor Trevino

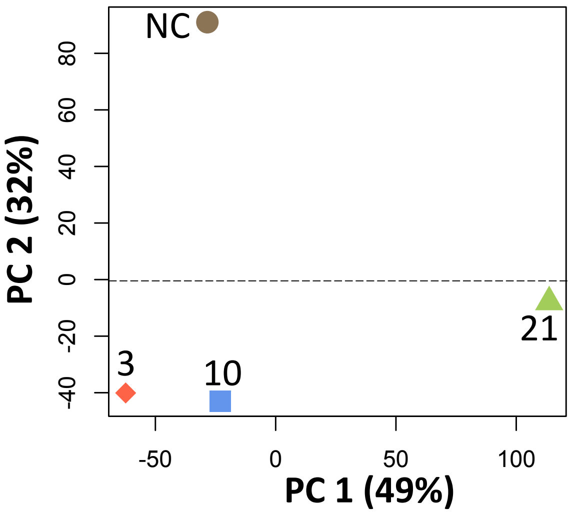

Figure S1. Principal components analysis of the overall gene expression profiling. Axes show first and second principal components along with variance explained. Numbers represent clones. NC – Negative Control.

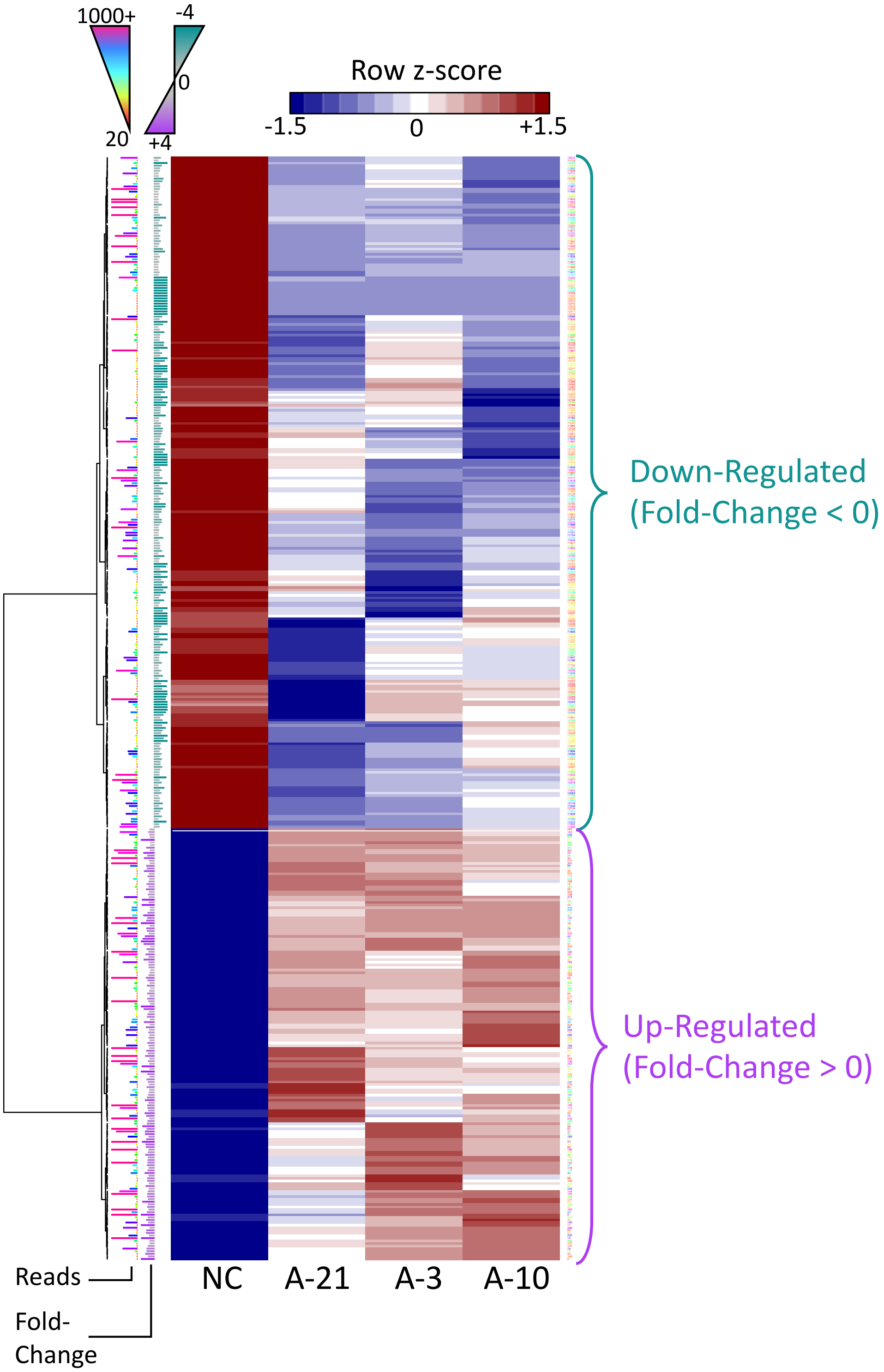

Figure S2. Heatmap of declared differential expressed genes. Rows show genes and columns show samples. Rows are scaled to z-score to clarify differences against negative control. Blue represents z-scores < -1 while Red represents z-scores > +1. “Reads” show the number of maximum reads per gene cut to 1,000 for clarity. Fold-Change shows the average fold-change from the three clones. NC – Negative Control, A-3, A-10, A-21 represent the three clones.
